# The effect of performance-based financing on illness, care-seeking and treatment among children: an impact evaluation in Rwanda

**DOI:** 10.1186/s12913-015-1033-7

**Published:** 2015-09-14

**Authors:** Martha Priedeman Skiles, Siân L. Curtis, Paulin Basinga, Gustavo Angeles, Harsha Thirumurthy

**Affiliations:** The Carolina Population Center, University of North Carolina at Chapel Hill, 400 Meadowmont Village Circle, 3rd Floor, Chapel Hill, NC 27517 USA; Department of Maternal and Child Health, Gillings School of Global Public Health, University of North Carolina, Chapel Hill, NC USA; Global Health Program, Bill and Melinda Gates Foundation, Seattle, WA USA; Rwanda Biomedical Center, Ministry of Health, Kigali, Rwanda; Department Health Policy and Management, Gillings School of Global Public Health, University of North Carolina, Chapel Hill, NC USA

## Abstract

**Background:**

Performance-based financing (PBF) strategies are promoted as a supply-side, results-based financing mechanism to improve primary health care. This study estimated the effects of Rwanda’s PBF program on less-incentivized child health services and examined the differential program impact by household poverty.

**Methods:**

Districts were allocated to intervention and comparison for PBF implementation in Rwanda. Using Demographic Health Survey data from 2005 to 2007–08, a community-level panel dataset of 5781 children less than 5 years of age from intervention and comparison districts was created. The impacts of PBF on reported childhood illness, facility care-seeking, and treatment received were estimated using a difference-in-differences model with community fixed effects. An interaction term between poverty and the program was estimated to identify the differential effect of PBF among children from poorer families.

**Results:**

There was no measurable difference in estimated probability of reporting illness with diarrhea, fever or acute respiratory infections between the intervention and comparison groups. Seeking care at a facility for these illnesses increased over time, however no differential effect by PBF was seen. The estimated effect of PBF on receipt of treatment for poor children is 45 percentage points higher (*p* = 0.047) compared to the non-poor children seeking care for diarrhea or fever.

**Conclusions:**

PBF, a supply-side incentive program, improved the quality of treatment received by poor children conditional on patients seeking care, but it did not impact the propensity to seek care. These findings provide additional evidence that PBF incentivizes the critical role staff play in assuring quality services, but does little to influence consumer demand for these services. Efforts to improve child health need to address both supply and demand, with additional attention to barriers due to poverty if equity in service use is a concern.

**Electronic supplementary material:**

The online version of this article (doi:10.1186/s12913-015-1033-7) contains supplementary material, which is available to authorized users.

## Background

In sub-Saharan Africa, where pneumonia, diarrhea, and malaria remain the leading killers for children under five, the mortality rate remains twice that of the global average [1]. Particularly vulnerable are children from the poorest households, who are 80 % more likely to die in the first five years of life compared to children from the wealthiest households [1]. Children from poorer families often have higher exposure to communicable and chronic diseases due to inadequate sanitation, insufficient drinking water, poor housing, and poor air quality, coupled with diminished resistance to disease due to malnutrition and micro-nutrient deficiencies [2]. Exacerbating this problem, in poorer communities health facilities are frequently understaffed, poorly equipped, and less well organized, resulting in health services being less responsive to the needs of the population [2].

Eight high impact preventive and curative child survival interventions have been identified as those with “the highest potential impact on child mortality” if universal coverage is achieved [3]. These interventions include four preventive efforts: measles and DTP immunizations; vitamin A supplements; and distribution of treated bednets, which all benefit from national campaigns that target all populations. The remaining four interventions, skilled attendant at delivery, professional care-seeking for pneumonia, use of oral rehydration for diarrhea, and treatment with antimalarials, typically rely on formal health facility services. The success of comprehensive facility-based interventions requires a base level of service use to have a measurable effect [4]. The question posed by this study is whether a supply-side financing system can increase the use of and improve the quality of facility-based child health services, particularly for the poor. Rwanda’s performance-based financing program offers a good opportunity to examine this question.

### Performance-based financing in Rwanda

In the late 1990s, the reintroduction of health service user fees in Rwanda led to a rapid decline in service utilization. The fixed salaries and standard bonus payment system for health providers, meanwhile, provided no financial incentive to maximize productivity or extend the reach of services to the populations in need [5, 6]. As a result, in 2005 the government adopted a national performance-based financing (PBF) program for health centers and hospitals. Performance-based financing (PBF) is a supply-side health financing initiative that aims to improve the quantity and quality of health service outputs through monetary incentives to facilities and/or providers. Rwanda’s PBF was designed to incentivize health facility personnel to increase access to facility services, strengthen health worker productivity, and improve service quality. PBF contracting with health facilities allowed the local health authorities to distribute these supplemental performance funds according to local priorities; typically provider bonuses or facility supplies and equipment.

Under these contracts, higher productivity was explicitly incentivized through a payment-per-service provided for 14 evidence-based primary maternal and child health services (Table 1). These services, a mix of preventive and curative care, were incentivized at different rates ranging from USD$4.59 per facility delivery and/or emergency transfer for obstetric care to USD$0.09 for the first prenatal care visit; curative care visits were one of the least incentivized services at only 18 cents per visit [7, 8]. Service quality was also rewarded based on a quarterly quality score created from monthly monitoring and assessment visits of select services. This quality score was used to weight the overall PBF payment, such that facilities received only a portion of the performance payment if the quality score was not perfect. Quality of growth monitoring visits was the most highly weighted component (0.52), followed by curative care; management of the facility was also included but with lower weights.

**Table 1:** Service outputs and quality weights used to determine performance-based financing payments

Service Outputs – payment per unit	Payment	Quality of services	Weight
Visit and Outreach	(USD)	Curative care	0.170
Facility delivery	4.59	Delivery	0.130
1st time family planning visits (new users)	1.83	Prenatal care	0.126
Completed childhood vaccines on time	0.92	Family planning	0.114
four completed ANC visits	0.37	HIV services	0.090
One-month contraceptive resupply	0.18	Immunizations	0.070
Curative care visits	0.18	Pharmacy management	0.060
Child (0–59 months) growth monitoring visits	0.18	Growth monitoring	0.052
1^st^ prenatal care visits	0.09	General administration	0.052
Content of Care		Financial management	0.050
Emergency transfers to hospitals for obstetric care	4.59	Lab services	0.030
At-risk pregnancies referred to hospital for delivery	1.83	TB services	0.028
Malnourished children referred for treatment	1.83	Cleanliness	0.028
Other emergency referrals during curative treatment	1.83		
Appropriate tetanus vaccine during ANC	0.46		
2^nd^ dose of malaria prophylaxis during ANC	0.46		

Evaluations of PBF in Rwanda and similar financing strategies elsewhere have focused on assessing the program’s impact on coverage of child preventive care services and pregnancy-related maternal care, [8–10] yet few rigorous studies have examined the effect of PBF on usage of child curative care services in a multivariate analysis with appropriate comparison groups, [7, 11] and no studies were found to have examined the effect on equity of child curative care use. Zeng and colleagues evaluated the impact of PBF on incentivized and non-incentivized primary care services in Haiti [12]. No effect on the probability of preventive (vaccination, vitamin A supplementation) or curative (treatment of pneumococcal or diarrhea) visits among 1–4 year olds was found, while the probability of infants receiving vaccination increased. Facility selection in Haiti may have introduced endogeneity; however econometric techniques were employed to control for potential selection bias. In the Philippines, Peabody and colleagues evaluated the effect of PBF on inpatient hospital care for sick children [9, 13]. They found improved quality of physician care in intervention hospitals yet no change in the rate of hospitalization between the intervention and comparison hospitals. Quality of care was assessed quarterly using vignettes presented to randomly selected physicians rather than direct observation or patient outcomes. In Rwanda, the phased implementation of the program enables identification of a suitable comparison population and the assessment and incentive strategy for quality of care provides a platform from which to measure quality of care across a spectrum of services, staff, and supplies.

The goal of this analysis was to estimate the effects of Rwanda’s PBF program on the prevalence of childhood illness, the care seeking behavior of households in response to these illnesses, and treatment received at health care facilities. More specifically, we explored the effect of PBF by household poverty, testing whether there was a narrowing of the equity gap in childhood illness and services received between children from the poorest and the least poor households.

## Methods

### Conceptual framework

Rwanda’s PBF incentives targeting prenatal care, facility delivery, immunizations, and growth monitoring were designed to provide the best start in life for infants and ensure their healthy development. Using population-based survey data, we first tested the hypothesis that PBF reduced the prevalence of childhood illness and examined whether the effect of PBF on illness was uniform across wealth status at a population level. Our second analysis tested the hypothesis that the implementation of PBF increased the probability of a caregiver seeking facility-based curative care services for a sick child. Lastly, among those who sought curative care, we tested the hypothesis that the probability of receiving medications and/or rehydration therapy was positively influenced by adoption of PBF.

### Study design

In 2005, the Government of Rwanda adopted a national PBF program with a phased implementation plan, facilitating an evaluation by the government in partnership with the World Bank [8]. Under the direction of the original program evaluators and concomitant with a Government decentralization and redistricting process, geographic areas nationwide were grouped on population density, rainfall and livelihood. Eight groups were created with on average 2–4 districts each; ten districts, among them the three districts surrounding Kigali, were excluded from this process due to previous PBF piloting. Districts within these eight groups were randomly allocated to early implementation between January 2006 and November 2007 (intervention) and to delayed implementation (comparison) beginning in April 2008 [8]. However, following this initial allocation, some facilities with prior PBF experience were found in the comparison group. For programmatic reasons the government requested uniform scale-up of PBF across a district, such that if a facility within a comparison district was already implementing PBF then the entire district should implement PBF. Thus the initial random allocation to early and delayed implementation had to be modified. Based on suspected early exposure to PBF, five districts with minimal exposure were reassigned from comparison to intervention group. One district was excluded from the evaluation due to extensive exposure. In summary nationwide, PBF was scaled-up in 12 early implementation districts, seven districts were allocated to late implementation, and 11 were excluded due to previous pilot work.

Health facility catchment areas mapped closely to the new administrative districts such that when an intervention district adopted PBF, the district population theoretically gained access to intervention sites. This design allowed for comparisons over time between the early implementers or intervention districts and delayed implementers or comparison districts. National household survey data, collected independently from the PBF intervention, provided pre- and post-implementation measures for selected child health outcomes.

### Data

Data from the 2005 Rwanda DHS (henceforth 2005) and the 2007–08 Rwanda Interim DHS (henceforth 2008) provided individual and household socio-demographic characteristics and health indicators for child health, including reported illness followed by care-seeking and treatment received for reported illness. The 2005 survey used a multi-stage national sampling frame and selected 462 primary sampling units (PSUs) [14] based on census enumeration areas, with field work completed from February 2005 through July 2005. A subset of 250 of these DHS 2005 PSUs were resampled for the 2008 DHS from December 2007 through April 2008 [15]. Geographic coordinates were available for 246 PSUs, facilitating the creation of a panel dataset of matched PSUs from 2005 and 2008. The 11 PBF pilot districts were excluded from the analysis. Longitudinal data from a total of 150 PSUs were thus used in the analysis, with 86 PSUs from the 12 intervention districts and 64 PSUs from the seven comparison districts.

The panel dataset included 5781 children less than 5 years of age at the time of each survey who lived in either an intervention (3307) or comparison district (2474). Slightly over half of the children were from the 2008 survey, 3157 (54.6 %).

Three primary outcomes were studied: prevalence of childhood illness, care-seeking at a health facility for reported illness, and treatment received among those who sought care at a facility. In this analysis, care-seeking was reported as success in actually being seen by a provider at a facility, therefore it does not include those who may have tried to see a provider at a facility and failed. Receipt of treatment among those who sought care was defined as receiving some medication for the condition. Data for reported cases of diarrhea, fever, and symptoms of acute respiratory infections (ARI), care sought for these episodes, and treatment received were collected in 2008; treatment information for ARI was not collected in 2005. Across both survey years, illness with diarrhea, fever, or ARI in the preceding two weeks was reported for fewer than 30 % of children; subsequent care-seeking was sought for fewer than 40 % of the ill children, effectively reducing the sample for the analysis of whether treatment was obtained. To maximize the data available, reported illnesses were combined as described below.

In the DHS, caregivers were asked if any child in the home was ill with diarrhea, fever, and/or a cough with short, rapid breathing (symptoms of ARI) in the previous two weeks. Responses were combined into two dichotomous illness variables: illness with diarrhea, fever and/or ARI; and illness with diarrhea and/or fever, excluding ARI. This allowed the creation of data subsets for those ill including ARI (*n* = 2073) and those ill excluding ARI (*n* = 1742). Questions regarding treatment received were asked only of the latter group in both survey years. Caregivers who reported a sick child were subsequently asked whether advice or treatment was sought from any source. All those who reported seeking advice from a public or private hospital, health center, clinic, or health post were coded as having sought care at a health facility. Among those seeking care for diarrhea and/or fever, a series of follow-up questions were also asked to identify any treatment or medications administered, either at home or a facility. A dichotomous variable for treatment received was constructed to indicate whether (a) a child with diarrhea received oral rehydration salts, was recommended home fluids, increased fluids, and/or antibiotics; or (b) if a child sick with fever received a fever reducer and/or an antimalarial. All other responses were coded a not having received treatment.

The key independent variables were residence in a PBF intervention district and household wealth quintile. Assignment to the PBF intervention group was based on the district in which the survey PSU was located; hence all children from the same PSU were assigned identical PBF status. Household wealth scores based on asset ownership and housing characteristics were created separately for households in the 2005 and 2008 study samples. Polychoric principal component analysis (PCA) was used to calculate a wealth score that maximized the contribution of binary and categorical variables [16]. The choice of assets for the wealth score was based on the economic context in Rwanda and data availability. Assets for 2005 included television, radio, telephone, bicycle, and land ownership; housing characteristics included electricity, drinking water, toilet facility, cooking fuel, and flooring material. Three assets were excluded due to perfect prediction with other assets: refrigerator, motorcycle, and car. For 2008, land ownership data was not collected, car and motorcycle ownership were combined as a single variable, and refrigerator was excluded, again for reasons of perfect prediction. The first component of the polychoric PCA was used to create the wealth index score, explaining 59 % of the variance for 2005 and 57 % for 2008. Households were divided into quintiles based on their wealth index score; the wealth quintile was assigned to each child living in the household.

### Empirical model

A difference-in-differences (DID) estimation strategy was used to evaluate the program effect of PBF on the three primary outcomes: probability of childhood illness, facility care-seeking and treatment received. The DID strategy estimates the change in outcome for the intervention and comparison groups over time and takes the difference between the two trends to determine the average effect of PBF, written as:

1 $$ DID=\left({Y}_{PBF08} - {Y}_{PBF05}\right)-\left({Y}_{Non-PBF08}-{Y}_{Non-PBF05}\right). $$

A linear probability model with individual, maternal, and household covariates included to reduce residual variance and improve efficiency was used for estimation. Community fixed effects were subsequently included to control for time-invariant unobserved community differences. The DID with community fixed effects specification is written as:

2 $$ {Y}_{ijt} = {\beta}_0+{\beta}_1{X}_{ijt}+{\beta}_2Y{08}_t+{\beta}_3\left(Y{08}_t\ast PB{F}_j\right) + {\mu}_j+{\varepsilon}_{ijt}, $$

where subscripted indexes were defined as *i* = individual, *j* = community or PSU, and *t* = time (2005 or 2008). Terms in the model include the vector of covariates (X), a dummy variable for time period 2007–08 (Y08 = post-implementation), and a dummy program variable for PSUs located in districts with performance based financing (PBF = 1 for intervention district, 0 for comparison). The primary coefficient of interest was β_3_, which captured the effect of the PBF program on the outcome Y. By subtracting the differences over time between program and non-program areas, the unobserved time-invariant community fixed effects (μ_j_) were differenced out.

To identify heterogeneous effects by poverty, the wealth quintiles were collapsed into a dichotomous variable with the two poorest quintiles groups together as poor and the two upper quintiles grouped as non-poor. Children from the middle wealth quintile were dropped from the model. Adding an interaction term between year, PBF, and poverty to the model allows one to examine the change in probability of an outcome across three dimensions: i) change in outcome over time, between 2005 and 2008; ii) change in outcome between the intervention and comparison groups; and iii) change in outcome between the poorest children and the least poor children. The model specification is shown below.

3 $$ \begin{array}{l}{Y}_{ijt} = {\beta}_0+{\beta}_1{X}_{ijt}+{\beta}_2Y{08}_t+{\beta}_3\left(Y{08}_t\ast PB{F}_j\right)+{\beta}_4\left(PO{V}_{ijt}\right)+{\beta}_5\left( PB{F}_j\ast PO{V}_{ijt}\right)+\\ {}{\beta}_6\left(Y{08}_t\ast PO{V}_{ijt}\right)+{\beta}_7\left(Y{08}_t\ast PB{F}_j\ast PO{V}_{ijt}\right) + {\mu}_j+{\varepsilon}_{ijt},\end{array} $$

where subscripted indexes were defined as *i* = individual, *j* = community, and *t* = time. A binary variable for poverty was added (POV = 1 for the poor; =0 for the non-poor). The primary coefficient of interest is the triple interaction term (β_7_), which captures the difference in the impact of the program between the poor and the non-poor. Due to concurrent scale-up of a national community based insurance program, interaction terms between insurance status and PBF residence, and insurance with wealth quintiles were also tested.

For each outcome a basic linear probability model (LPM) and an LPM with community fixed effects were estimated. Robust standard errors were clustered at the district level where treatment was assigned. The LPMs with fixed effects were used to calculate the DID and DID with and without the poverty interaction. The full basic and fixed effects models are presented in appendices A and B [see Additional file 1]. Lastly, the fixed effects models were run with and without “choice” variables (insurance, prior facility delivery) to identify potential bias in estimates due to inclusion of these variables that may arguably introduce endogeneity to the model. No significant or substantial differences in program effect were found with or without these choice variables.

The study, based exclusively on secondary analyses of publicly-available data, was reviewed and deemed exempt by the University of North Carolina (UNC) Institutional Review Board. All analyses were completed in Stata SE 11.2 (StataCorp, College Station, TX).

## Results

There were no significant differences in the observed characteristics of the intervention and comparison groups at baseline (Table 2). In the 2005 survey, the reported prevalence of diarrhea, fever and ARI was 15.6, 25.1, and 18.2 % respectively in the comparison group (Table 3). Facility consultation among the comparison group ranged from 12 to 17 % in 2005, and treatment received ranged from 30 to 62 %. No statistically significant differences between the intervention and comparison groups were found in the prevalence of these illnesses, the care sought, or the treatment received.

**Table 2:** Comparison of child, mother, and household characteristics between the intervention and comparison samples at baseline, 2005 DHS weighted data

	Total		Intervention		Comparison		Difference	
	(*N* = 2619)	%	(*N* = 1631)	%	(*N* = 988)	%	Perc.Pt^a^	p-value^b^
Child								
Age <12 months	590	22.5	376	23.0	214	21.6	1.40	0.406
Sex: Boy	1333	50.9	843	51.7	489	49.5	2.20	0.289
Birth order: 1st birth	439	16.7	275	16.9	164	16.5	0.40	0.846
Birth order: ≥ 5th	988	37.7	632	38.7	356	36.0	2.70	0.335
Slept under bednet	277	10.8	179	11.2	97	10.1	1.10	0.621
Health facility birth	586	22.4	348	21.4	238	24.1	−2.70	0.409
Mother								
Age <20 years	497	19.0	336	20.6	161	16.3	4.30	0.064
Age ≥35 years	386	14.7	239	14.6	147	14.9	−0.30	0.886
Primary school grad.	445	17.0	275	16.8	170	17.2	−0.40	0.881
Married	1310	88.2	1429	87.6	881	89.1	−1.50	0.380
Previous child death	1126	43.0	714	43.8	412	41.7	2.10	0.495
Household								
Wealth status								
Poorest	498	19.0	318	19.5	180	18.2	1.30	0.619
Poorer	534	20.4	296	18.1	238	24.1	−6.00	0.050
Middle	548	20.9	367	22.5	181	18.3	4.20	0.109
Less poor	525	20.0	342	21.0	182	18.4	2.60	0.326
Least poor	515	19.6	308	18.9	206	20.9	−2.00	0.608
Health insurance	1283	49.0	771	47.3	512	51.8	−4.50	0.364
Rural residence	2399	91.6	1523	93.4	876	88.6	4.80	0.254
Improved toilet^c^	610	23.3	338	20.7	272	27.5	−6.80	0.077
Clean water source^d^	871	33.2	601	36.9	269	27.3	13.50	0.066

^a^Percentage point difference between intervention and comparison groups

^b^T-tests comparing proportions between intervention and comparison groups

^c^Includes flush toilets and improved latrines

^d^Includes tap water and water from improved wells

**Table 3:** Number and percent of children reported ill, seeking care, and receiving treatment in past 2 weeks by study sample and year

	Intervention group					Comparison group				
	2005^a^		2008^b^		Absolute change^c^	2005^a^		2008^b^		Absolute change^c^
	N	%	N	%	%	N	%	N	%	%
Reported illness										
Diarrhea	218	13.4	270	13.4	0.0	154	15.6	151	12.4	−3.2
Fever	437	26.8	426	21.2	−5.6*	247	25.1	276	22.7	−2.4
ARI	305	18.9	376	18.7	−0.2	177	18.2	212	17.5	−0.7
Facility consultation among those sick										
Diarrhea	35	16.4	87	32.5	16.1**	18	12.0	36	23.7	11.7*
Fever	108	24.8	146	35.7	10.9*	66	27.0	96	35.7	8.7
ARI	90	29.4	98	27.1	−2.3	47	27.0	70	35.3	8.3
Treatment among those who received facility consultation^d^										
For diarrhea:										
ORT	22	61.9	56	64.4	2.5	11	61.8	26	73.0	11.2
Antibiotics	18	52.1	23	26.8	−25.3	9	49.9	11	29.9	−20.0
For fever:										
Fever reducer	46	42.4	73	50.5	8.1	29	46.6	43	45.4	−1.2
Antimalarial	22	20.4	16	11.3	−9.1	19	29.6	14	14.7	−14.9

Denominators (not shown) change by study sample, year, and outcome

^a^T-tests found no statistical difference between the intervention and comparison groups at baseline

^b^T-tests found no statistical difference between the intervention and comparison groups post-intervention

^c^T-tests for differences between 2005 and 2008; *p < 0.05, **p < 0.01

^d^Insufficient numbers to calculate test statistic

Comparison of the absolute change in reported prevalence of diarrhea, fever, and ARI from 2005 to 2008 shows a decline in prevalence in both the intervention and comparison groups, although the decline was significant only for fever in the intervention group (−5.6 percentage points, *p* = 0.018) (Table 3). Change in care-seeking behavior and treatment received was heterogeneous across groups and reported illness. Use of ORT increased from 2005 to 2008, while use of antibiotics declined dramatically, though the number of observations is limited. This decline may be attributable to the seasonality of dysentery which is more prevalent during the rainy season, when 2005 data was collected, versus the dry season, when 2008 data was collected. Among those with fever, fever reducers were more commonly taken compared to antimalarial medications. Use of antimalarials in fact declined from 2005 to 2008 for both the intervention and comparison groups, which may have been due to a lower national prevalence of malaria [17].

There was no measurable difference between the intervention and comparison groups in the estimated change in probability of reporting illness with diarrhea, fever and/or ARI (DID = −0.050), nor for reporting diarrhea and/or fever only (DID = −0.028) (Table 4). Seeking care at a facility for these illnesses increased over time for both disease groups by approximately 7 percentage points, however no differential effect by PBF was seen (Additional file 1).

**Table 4:** Estimated effects of PBF on reported illness, facility care-seeking, and treatment received among children under 5 years of age, from 2005 to 2008: DID Estimates

	Diarrhea, Fever and/or ARI			Diarrhea and/or Fever		
	N	DID coeff^a^	(SE)^b^	N	DID coeff^a^	(SE)^b^
Reported illness^c^	4501	−0.050	(0.066)	4501	−0.028	(0.060)
Facility care-seeking^d^	1606	−0.081	(0.058)	1355	−0.072	(0.062)
Treatment received^d^	--	--	--	399	0.221	(0.113)

^a^Coefficient for the DID term (β_3_ in Equation 2)

^b^Robust standard errors in parentheses

^c^LPM with fixed effects, covariates include: child’s age, birth order, gender, and facility birth; mother’s age, education, marital status; household wealth, toilet facilities, drinking water source, and bednet use

^d^LPM with fixed effects, covariates include: child’s age, birth order, gender, and facility birth; mother’s age, education, marital status; household wealth, insurance status, and previous child death

Data on medical treatments received were only available for those who reported an episode of diarrhea and/or fever in the prior 2 weeks. The average estimated PBF program effect on receipt of treatment, conditional on seeking treatment at a facility, was 0.221 (*p* = 0.065), suggesting a program effect on the quality of services provided in PBF districts. For this outcome, the basic LPM without fixed effects produced smaller effects, smaller standard errors, and a smaller overall DID estimate. This is a departure from the pattern established for the illness and care-seeking where the estimates were close in size and typically slightly over-estimated in the basic LPM. One possible explanation is that the quality of services may be more strongly influenced by the rural location, which is differenced out of the fixed effects estimation. When a rural dummy variable is included in the basic LPM, rural residence is predictive of treatment (β = 0.163, *p* = 0.003), conditional on seeking treatment.

Including a poverty interaction term allowed us to examine the effect of PBF on equity of reported illness. No differences in reported illness were found between the poor and non-poor relative to PBF implementation (Table 5). Estimates for care seeking relied on the sample of reportedly ill children. The use of a Heckman selection model was considered but ruled out as no selection bias for the subsample of ill children was found (i.e., the probability of reported illness was not associated with the PBF intervention). No significant difference in care-seeking behavior by poverty and relative to PBF intervention was estimated.

**Table 5:** Estimated effects of PBF on reported illness, facility care-seeking, and treatment received among children under 5 years of age by poverty, from 2005 to 2008: most poor compared to the least poor (referent group)

	Diarrhea, Fever and/or ARI			Diarrhea and/or Fever		
	N	Interaction coeff^a^	(SE)^b^	N	Interaction coeff^a^	(SE)^b^
Reported illness^c^	4501	0.056	(0.071)	4501	0.061	(0.070)
Facility care-seeking^d^	1606	0.109	(0.073)	1355	0.113	(0.094)
Treatment received^d^	--	--	--	399	0.446*	(0.210)

*p < 0.05

^a^Coefficient for the poverty interaction term (β_7_ in Equation 3)

^b^Robust standard errors in parentheses

^c^LPM with fixed effects, covariates include: child’s age, birth order, gender, and facility birth; mother’s age, education, marital status; household wealth, toilet facilities, drinking water source, and bednet use

^d^LPM with fixed effects, covariates include: child’s age, birth order, gender, and facility birth; mother’s age, education, marital status; household wealth, insurance status, and previous child death

The impact on equity of child services is most apparent when studying the effect on treatment received in PBF districts. Children from the poor households in PBF districts had a 44.6 percentage point higher estimated probability of receiving medicine (*p* = 0.047) compared to children from the non-poor households. This pro-poor effect is even larger (β = 0.692, *p* = 0.002) when the five minimally pre-study exposed districts are excluded from the analysis (data not shown). This suggests that the full-sample estimate produces a diluted program effect due to some earlier exposure.

## Discussion

The PBF program in Rwanda was designed to improve facility-based primary maternal and child health services, thereby reducing morbidity and mortality among vulnerable populations. In our analysis, however, we found no evidence to support the hypothesis that PBF districts experienced decreased morbidity from diarrhea, fever, or symptoms of ARI relative to the comparison districts. Moreover there was no finding of a differential effect by household poverty; that is PBF was neither a pro-poor nor a pro-rich strategy for reducing childhood illness. This is in line with findings from Schellenberg and colleagues, where no association between economic status and childhood morbidity was found in a population-based household survey in Tanzania [18].

There are several possible explanations for this finding. First the window of time between initial program payments in June 2006 and the beginning of the follow-up survey in December 2007 may arguably be too short to observe an impact on childhood health from better prenatal care, safer deliveries, and improved growth monitoring. Second, disease prevalence declined nationally likely due to multiple factors. For example, malaria burden decreased dramatically following a national insecticide-treated bednet campaign in September-October 2006. The prevalence of malaria in Rwanda in 2007 was half of that reported in 2005 [17]. The seasonality of data collection in 2005 compared to 2008 was likely correlated with seasonality of disease, particularly for severe diarrhea and malaria. Third, acute childhood illnesses, particularly diarrhea and pneumonia, are the result of environmental exposures to water and air quality or crowding. PBF was not designed to change the home environment or vectors for these diseases; rather the focus of PBF was on increasing the use and quality of health facility services.

The theoretical argument for improved health service outputs in PBF program areas is conceptually stronger. Do provider incentives increase the proportion of sick children seeking outpatient curative care visits? Or conversely, does a low incentive for curative services adversely affect care seeking? No PBF program effect was found for facility care-seeking among those with diarrhea, fever and/or ARI, or just diarrhea and/or fever. Living in a household with someone who had health insurance and being born in a health facility were associated with a higher probability of seeking facility care, which may be indicative of an underlying propensity to use the health system (appendices). One could argue that these choice variables are endogenous and may cause the results to be biased, [19] however when the models were run without these choice variables, no differences in outcomes were found.

An alternate interpretation of our results for care-seeking behavior is that the dramatic increase in health insurance in Rwanda increased economic access to services such as curative consultations for families in intervention and comparison districts regardless of propensity to use services. Community based health insurance or *mutuelles de santé* (*mutuelles*) were developed in Rwanda in an effort to reduce the financial barriers and risks families faced with unexpected medical costs and to mobilize resources locally for health facilities. By 2007, an estimated 68 % of households had at least one member covered by health insurance, 96 % of these participated in a *mutuelle* [15]. In a 2012 evaluation of Rwanda *mutuelles* by Lu and colleagues, the use of curative care consultations among ill children with *mutuelles* insurance was double the rate among those without insurance [20]. This reinforces similar findings of increased service use among the insured in Rwanda [21–23]. Interactions between insurance and PBF and insurance and wealth quintiles were tested but not included due to insignificance. Disentangling the effects of PBF from the rise of *mutuelles* is a difficult but promising area for future study [23].

The quantity of curative care consultations was incentivized through PBF at one of the lowest rates (USD$0.18 per visit), yet the quality of curative care was promoted through the quality assessment. The quality assessment covered multiple maternal and child health care services and employed a series of observations, visual inspections, review of records, and some provider and patient interviews. Of interest to this study, direct observation of primary care consultations for children less than 5 years of age and an inventory of essential medicines and products were included in the assessment protocol. Points were awarded to facilities based on the adequacy of the different services, supplies and facility environment observed. The average quarterly assessment score served as the weight for the final quarterly PBF incentive payment [24]. Quality of curative care services was the most heavily weighted item in the monthly quality assessment score and pharmacy management contributed an additional mid-level weight. So even though outpatient consultations were one of the least incentivized services, the quality score was influenced by care provided and pharmacy supplies. This incentive design minimized the limited patient outreach role played by providers but rewarded providers and facilities for their critical role in assuring the quality of treatment provided. The question is whether this quality assessment embedded in the PBF incentive structure affected the treatment received by sick children.

The data suggest that children living in PBF districts were more likely to receive medications and/or ORT when seeking facility care for illness in 2008 relative to children living in comparison districts, although this finding was not statistically significant. However this finding masks the heterogeneous effects by poverty. The poorest children in PBF districts benefitted more relative to the non-poor in PBF districts and to those living in comparison districts. In intervention districts, the quality assessments included a review of pharmacy stock which likely motivated facilities to maintain an adequate supply of products. This finding provides further evidence to support the notion put forward by Basinga and colleagues that those services within the control of the provider and less reliant on repeated initiation by the consumer, were more likely to improve under PBF [8]. Granted not all cases of diarrhea or fever require a medical intervention, however Boerma and colleagues found in a multi-country study that the treatment of sick children was the most underutilized service [25]. Huntington and colleagues found a decrease in prescribed treatment for children seeking curative care in pay-for-performance intervention sites in Egypt; however, among those prescribed treatment the probability of receiving medication during the visit was significantly higher compared to those seen in comparison sites [11]. Globally, universal coverage of oral rehydration therapy could prevent an estimated 15 % of deaths under age 5, while antimalarials and treatment of pneumonia could prevent an additional 5 % each [26]. Given the exceptionally low usage for antimalarials in Rwanda, 5.6 % among children under 5 years, and the low ORT use (39 %), one can make the case for improving treatment rates through the improved supply of medications [15].

### Limitations

Despite efforts by the original PBF evaluators to randomly allocate districts to intervention and comparison groups, the reassignment of six districts was required due to practical considerations of prior PBF implementation. However the DID estimation strategy used in this study controlled for unobserved, time-invariant differences across the final intervention and comparison districts that may have resulted from this reassignment. Additionally, analyses run excluding these districts produced similar findings overall and stronger effects for treatment among the poorest (data not shown).

The potential contamination of study findings by other interventions or health system changes is a challenge for impact evaluations and is one of the limitations of this study. Econometric techniques were employed to control for potential unobserved time-invariant confounders such as community or facility infrastructure. Potential bias remains, however, if unmeasured shocks to the health system occurred for some and not all study districts, particularly if an intervention was targeted to build on the new PBF system. For example, if the President’s Malaria Initiative substantially increased the availability of artemisinin-based combination therapy for the treatment of malaria only in intervention districts, perhaps due to a perception by donors of better pharmaceutical supply chains in these districts, then results may be biased. While these scenarios may confound program effects, the alternative of withholding interventions for the duration of a study is impractical given the multiple health challenges faced in developing countries.

An additional limitation was the timing of data collection for the DHS in 2005 and 2008. Seasonality has epidemiologic implications for the diseases studied, hence the mismatch between seasonal data collection in 2005 and 2008 likely contributed to the childhood illness reported. However this should not bias any observed intervention effects given that data were collected during the same seasons in intervention and comparison districts.

## Conclusions

In the context of Rwanda, these evaluation findings support the hypothesis that incentivizing quality of care improves quality among the poorest children. The incentive structure rewards the critical role providers play in assuring the quality of services provided. Yet an increase in care seeking for routine childhood illness was not realized under the supply-side incentive program. Looking beyond the Rwanda experience, the findings from this study should encourage the design of PBF programs with full recognition of the relative strengths and weaknesses of the system. First, health care providers will have the most influence on the quality of care and treatment provided once someone seeks care at a facility. Hence, prescribing the correct treatment or providing adequate nutrition screening and immunizations to children is within the purview of the facility and should be a part of any supply-side PBF scheme.

Recruiting families into care is more difficult to do without addressing demand-side constraints. Consumer inputs designed to reduce financial barriers, such as insurance, or to increase demand through outreach and education by community health worker (CHW) programs, are necessary companion strategies to supply-side efforts. While evidence supports the use of CHWs to reduce childhood morbidity and mortality from routine illness, [27] there are no rigorous evaluations of the effect of incentivizing CHWs through PBF for child health. In a PBF scheme, CHWs could be incentivized to educate and refer the population to available child health care services. Alternately, CHWs may be trained to provide some doorstep services for those families where travel is a barrier to facility care. These services can cover rapid testing and in-home treatment of childhood illnesses such as diarrhea, fever or ARI. Implementing a network of CHWs requires comprehensive training in patient education, appropriate in-home services, and recognizing signs and symptoms for referral care. Moreover, controls need to be put in place to monitor the quality of this off-site work as well as the validity of reporting. An evaluation of Rwanda’s more recent experience with expanding PBF to CHWs, will be a welcome addition to the evidence base for PBF.

To address inequity in service use a targeted approach is needed. Two targeting methods, geographic and needs-based, deserve consideration. A geographic approach has been successful in countries where underserved populations are identifiable based on location, such as rural remote communities or poor, urban slums [28, 29]. The PBF incentive structure can be designed to favor service provision in these communities through higher incentives per service or combined with consumer vouchers to encourage use. A needs-based approach could either incentivize services provided only to the eligible poorer population, as seen in Bangladesh, [30] or could set differential performance targets that favor services provided to the poor. Both methods oblige health facilities to identify clients by wealth status, establish outreach efforts to recruit clients from poorer households, and reduce consumer barriers for these poorest families. In Rwanda PBF targeting could build on existing government insurance subsidy programs for the poorest 25 % of the population. An equity performance target could incentivize services provided to these beneficiaries of subsidized insurance. Another program gaining traction in some countries is the issuance of identification cards for poor households to provide access to certain subsidized services. Access and reimbursement for health services could become a more prominent feature in these efforts.

As countries across sub-Saharan Africa strive to reduce childhood mortality, efforts to improve diagnosis and treatment for the leading childhood killers are being tested and evaluated. New PBF strategies are gaining international attention as a potential financing mechanism that will improve the scope and reach of services to all families. Understanding the provider’s role and scope of influence vis-à-vis access and quality will inform policy makers who advocate for and implement change.

## Additional file

Additional file 1: Table S1. Linear probability models for effect of PBF on reported diarrhea, fever and/or symptoms of ARI, and facility care-seeking, differentiated by poverty. **Table S2**. Linear probability models for effect of PBF on reported diarrhea and/or fever, facility care-seeking, and treatment received, differentiated by poverty. (DOCX 44 kb)

## Abbreviations

ANC: Antenatal care
ARI: Acute respiratory infection
CHW: Community health worker
DDD: Difference-in-difference-in-differences
DHS: Demographic and Health Survey
DID: Difference-in-differences
LPM: Linear probability model
PBF: Performance-based financing
PCA: Principal component analysis
PSU: Primary sampling unit
UNC: University of North Carolina
