# Supplementary material for: The effect of performance-based financing on illness, care-seeking and treatment among children: an impact evaluation in Rwanda
Source: BMC Health Serv Res. 2015 Sep 14;15:375. doi: 10.1186/s12913-015-1033-7 (PMC4570690; doi:10.1186/s12913-015-1033-7)
Supplement: Additional file 1: Table S1. — Linear probability models for effect of PBF on reported diarrhea, fever and/or symptoms of ARI, and facility care-seeking, differentiated by poverty. Table S2. Linear probability models for effect of PBF on reported diarrhea and/or fever, facility care-seeking, and treatment received, differentiated by poverty. (DOCX 44 kb) [file 12913_2015_1033_MOESM1_ESM.docx]

Table S1 Linear probability models for effect of PBF on reported diarrhea, fever and/or symptoms of ARI, and facility care-seeking, differentiated by poverty

|  | **Reported Diarrhea, Fever, ARI** | | | | | | | |  | | **Facility Care-Seeking** | | | | | | | | | | | |  |  |
| --- | --- | --- | --- | --- | --- | --- | --- | --- | --- | --- | --- | --- | --- | --- | --- | --- | --- | --- | --- | --- | --- | --- | --- | --- |
|  | **DID** | | **DID**  **with FE** | | | **DID with Poverty Interaction, FE** | | |  | | **DID** | | **DID**  **with FE** | | | | | | | **DID with Poverty Interaction, FE** | | |  |  |
| Year 2008 | 0.036 | | 0.036 | | | 0.07 | | |  | | 0.097** | | | | | 0.098* | | | | 0.151* | | | |  |
|  | (0.057) | | (0.058) | | | (0.046) | | |  | | (0.035) | | | | | (0.043) | | | | (0.065) | | | |  |
| PBF District | 0.028 | |  | | |  | | |  | | 0.028 | | | | |  | | | |  | | | |  |
|  | (0.050) | |  | | |  | | |  | | (0.037) | | | | |  | | | |  | | | |  |
| 2008 * PBF District | -0.047 | | -0.05 | | | -0.078 | | |  | | -0.064 | | | | | -0.081 | | | | -0.134 | | | |  |
|  | (0.064) | | (0.066) | | | (0.061) | | |  | | (0.054) | | | | | (0.058) | | | | (0.080) | | | |  |
| 2008 * PBF * Most Poor | | |  | | | 0.055 | | |  | |  | | | | |  | | | | 0.109 | | | |  |
|  |  | |  | | | (0.071) | | |  | |  | | | | |  | | | | (0.073) | | | |  |
| 2008 * Most Poor |  | |  | | | -0.074 | | |  | |  | | | | |  | | | | -0.112 | | | |  |
|  |  | |  | | | (0.050) | | |  | |  | | | | |  | | | | (0.065) | | | |  |
| PBF*Most Poor |  | |  | | | 0.007 | | |  | |  | | | | |  | | | | 0.002 | | | |  |
|  |  | |  | | | (0.052) | | |  | |  | | | | |  | | | | (0.061) | | | |  |
| Most Poor | -0.01 | | -0.008 | | | 0.012 | | |  | | -0.082* | | | | | -0.059 | | | | -0.034 | | | |  |
|  | (0.030) | | (0.031) | | | (0.038) | | |  | | (0.032) | | | | | (0.035) | | | | (0.048) | | | |  |
| Rural Residence | 0.014 | |  | | |  | | |  | | 0.083* | | | | |  | | | |  | | | |  |
|  | (0.023) | |  | | |  | | |  | | (0.037) | | | | |  | | | |  | | | |  |
| Health Insurance |  | |  | | |  | | |  | | 0.158*** | | | | | 0.129*** | | | | 0.130*** | | | |  |
|  |  | |  | | |  | | |  | | (0.025) | | | | | (0.025) | | | | (0.026) | | | |  |
| Child's Age (Ref: 0-11 months) | | | | | | | | | | | | | | | | | | | | | | | |  |
| 12-23 months | 0.083*** | | 0.087*** | | | 0.087*** | | |  | | -0.018 | | | | | -0.022 | | | | -0.022 | | | |  |
|  | (0.014) | | (0.014) | | | (0.014) | | |  | | (0.034) | | | | | (0.034) | | | | (0.033) | | | |  |
| 24-35 months | -0.034* | | -0.030* | | | -0.030* | | |  | | -0.013 | | | | | -0.002 | | | | -0.001 | | | |  |
|  | (0.014) | | (0.013) | | | (0.013) | | |  | | (0.051) | | | | | (0.053) | | | | (0.054) | | | |  |
| 36-47 months | -0.092*** | | -0.088*** | | | -0.087*** | | |  | | -0.064 | | | | | -0.073 | | | | -0.071 | | | |  |
|  | (0.016) | | (0.016) | | | (0.016) | | |  | | (0.040) | | | | | (0.037) | | | | (0.037) | | | |  |
| 48-59 months | -0.124*** | | -0.122*** | | | -0.121*** | | |  | | -0.115* | | | | | -0.112* | | | | -0.113* | | | |  |
|  | (0.016) | | (0.016) | | | (0.017) | | |  | | (0.049) | | | | | (0.052) | | | | (0.051) | | | |  |
| Birth Order (Ref: 5th or higher) | | | | | | | | | | | | | | | | | | | | | | | |  |
| First | -0.014 | | -0.016 | | | -0.016 | | |  | | 0.054 | | | | | 0.06 | | | | 0.059 | | | |  |
|  | (0.038) | | (0.039) | | | (0.038) | | |  | | (0.049) | | | | | (0.046) | | | | (0.046) | | | |  |
| Second-Fourth | -0.036 | | -0.037 | | | -0.036 | | |  | | 0.032 | | | | | 0.031 | | | | 0.031 | | | |  |
|  | (0.027) | | (0.027) | | | (0.027) | | |  | | (0.038) | | | | | (0.039) | | | | (0.039) | | | |  |
| Child's Sex: Boy | 0.001 | | 0.001 | | | 0.000 | | |  | | 0.013 | | | | | 0.021 | | | | 0.018 | | | |  |
|  | (0.011) | | (0.011) | | | (0.011) | | |  | | (0.019) | | | | | (0.021) | | | | (0.021) | | | |  |
| Born in Facility | -0.011 | | -0.005 | | | -0.004 | | |  | | 0.121*** | | | | | 0.096*** | | | | 0.097*** | | | |  |
|  | (0.017) | | (0.017) | | | (0.017) | | |  | | (0.023) | | | | | (0.024) | | | | (0.024) | | | |  |
| Mother's Age | -0.003* | | -0.003 | | | -0.003 | | |  | | -0.001 | | | | | 0.000 | | | | 0.000 | | | |  |
|  | (0.002) | | (0.002) | | | (0.002) | | |  | | (0.002) | | | | | (0.003) | | | | (0.003) | | | |  |
| Mother's Education (Ref: No School) | | | | | | | | | | | | | | | | | | | | | | | |  |
| Primary School | -0.034 | | -0.034 | | | -0.034 | | |  | | 0.003 | | | | | -0.002 | | | | -0.003 | | | |  |
|  | (0.019) | | (0.019) | | | (0.018) | | |  | | (0.026) | | | | | (0.027) | | | | (0.028) | | | |  |
| Secondary School | -0.002 | | 0.002 | | | 0.003 | | |  | | 0.145*** | | | | | 0.160*** | | | | 0.162*** | | | |  |
|  | (0.025) | | (0.024) | | | (0.024) | | |  | | (0.032) | | | | | (0.031) | | | | (0.033) | | | |  |
| Mother Married | -0.031 | | -0.032 | | | -0.031 | | |  | | -0.037 | | | | | -0.036 | | | | -0.036 | | | |  |
|  | (0.017) | | (0.017) | | | (0.017) | | |  | | (0.037) | | | | | (0.048) | | | | (0.048) | | | |  |
| Improved Sanitation | 0.01 | | 0.007 | | | 0.014 | | |  | |  | | | | |  | | | |  | | | |  |
|  | (0.017) | | (0.018) | | | (0.018) | | |  | |  | | | | |  | | | |  | | | |  |
| Clean Water Source | -0.029 | | -0.031 | | | -0.031 | | |  | |  | | | | |  | | | |  | | | |  |
|  | (0.030) | | (0.033) | | | (0.032) | | |  | |  | | | | |  | | | |  | | | |  |
| Slept under Bednet | -0.004 | | -0.002 | | | -0.002 | | |  | |  | | | | |  | | | |  | | | |  |
|  | (0.017) | | (0.018) | | | (0.018) | | |  | |  | | | | |  | | | |  | | | |  |
| Previous Child Death | | |  | | |  | | |  | | 0.029 | | | | | 0.022 | | | | 0.022 | | | |  |
|  |  | |  | | |  | | |  | | (0.030) | | | | | (0.031) | | | | (0.031) | | | |  |
| Constant | 0.531*** | | 0.560*** | | | 0.547*** | | |  | | 0.106 | | | | | 0.187 | | | | 0.178 | | | |  |
|  | -0.082 | | -0.081 | | | -0.084 | | |  | | (0.099) | | | | | (0.115) | | | | (0.118) | | | |  |
| Number of Clusters | | 150 | 150 | | | 150 | |  | | 150 | | | | | 150 | | | | 150 | | |  |  |  |
| Number of Children | | 4501 | | 4501 | 4501 | | |  | | 1606 | | | | 1606 | | | | 1606 | | | |  |  |  |
| * p<0.05, **p<0.01, *** p<0.001 | | | | | | |  | | | | |  | |  | | |  | | | |  | | | |

Table S2 Linear probability models for effect of PBF on reported diarrhea and/or fever, facility care-seeking, and treatment received, differentiated by poverty

|  | **Ill with Diarrhea or Fever** | | |  | **Facility Care-Seeking** | | |  | | **Treatment Received** | | | | | | |  |
| --- | --- | --- | --- | --- | --- | --- | --- | --- | --- | --- | --- | --- | --- | --- | --- | --- | --- |
|  | **DID** | **DID with FE** | **DID with Poverty Interaction, FE** |  | **DID** | **DID with FE** | **DID with Poverty Interaction, FE** |  | | **DID** | | **DID with FE** | | **DID with Poverty Interaction, FE** | | |  |
| Year 2008 | -0.011 | -0.011 | 0.028 |  | 0.103** | 0.114* | 0.156 | |  | | -0.019 | | -0.089 | | -0.022 | | |
|  | (0.052) | (0.053) | (0.043) |  | (0.037) | (0.045) | (0.075) | |  | | (0.045) | | (0.092) | | (0.076) | | |
| PBF District | 0.010 |  |  |  | 0.024 |  |  | |  | | -0.089 | |  | |  | | |
|  | (0.047) |  |  |  | (0.038) |  |  | |  | | (0.064) | |  | |  | | |
| 2008 * PBF District | -0.025 | -0.028 | -0.060 |  | -0.039 | -0.072 | -0.124 | |  | | 0.094 | | 0.221 | | 0.069 | | |
|  | (0.059) | (0.060) | (0.055) |  | (0.059) | (0.062) | (0.093) | |  | | (0.081) | | (0.113) | | (0.138) | | |
| 2008 * PBF * Most Poor | |  | 0.061 |  |  |  | 0.113 | |  | |  | |  | | 0.446* | | |
|  |  |  | (0.070) |  |  |  | (0.094) | |  | |  | |  | | (0.210) | | |
| 2008 * Most Poor |  |  | -0.084 |  |  |  | -0.094 | |  | |  | |  | | -0.232 | | |
|  |  |  | (0.043) |  |  |  | (0.073) | |  | |  | |  | | (0.116) | | |
| PBF*Most Poor |  |  | -0.001 |  |  |  | 0.039 | |  | |  | |  | | -0.318 | | |
|  |  |  | (0.057) |  |  |  | (0.062) | |  | |  | |  | | (0.170) | | |
| Most Poor | -0.022 | -0.022 | 0.006 |  | -0.067* | -0.048 | -0.054 | |  | | -0.097* | | -0.103** | | 0.072 | | |
|  | (0.028) | (0.030) | (0.037) |  | (0.030) | (0.034) | (0.038) | |  | | (0.038) | | (0.034) | | (0.088) | | |
| Rural Residence | 0.033 |  |  |  | 0.087* |  |  | |  | | 0.163** | |  | |  | | |
|  | (0.023) |  |  |  | (0.043) |  |  | |  | | (0.055) | |  | |  | | |
| Health Insurance |  |  |  |  | 0.159*** | 0.118*** | 0.120*** | |  | | -0.132* | | -0.153 | | -0.146 | | |
|  |  |  |  |  | (0.030) | (0.030) | (0.030) | |  | | (0.065) | | (0.081) | | (0.077) | | |
| Child's Age (Ref: 0-11 months) | |  |  |  |  |  |  | |  | |  | |  | |  | | |
| 12-23 months | 0.080*** | 0.085*** | 0.086*** |  | -0.033 | -0.026 | -0.023 | |  | | -0.020 | | -0.045 | | -0.043 | | |
|  | (0.020) | (0.020) | (0.020) |  | (0.034) | (0.036) | (0.035) | |  | | (0.062) | | (0.060) | | (0.059) | | |
| 24-35 months | -0.028* | -0.025* | -0.025* |  | 0.000 | 0.023 | 0.024 | |  | | 0.055 | | 0.045 | | 0.051 | | |
|  | (0.012) | (0.011) | (0.012) |  | (0.047) | (0.047) | (0.048) | |  | | (0.065) | | (0.066) | | (0.069) | | |
| 36-47 months | -0.085*** | -0.080*** | -0.080*** |  | -0.079 | -0.086 | -0.083 | |  | | -0.040 | | -0.027 | | -0.021 | | |
|  | (0.020) | (0.019) | (0.019) |  | (0.043) | (0.043) | (0.042) | |  | | (0.075) | | (0.077) | | (0.076) | | |
| 48-59 months | -0.124*** | -0.121*** | -0.121*** |  | -0.126** | -0.115* | -0.114* | |  | | -0.174 | | -0.148 | | -0.154 | | |
|  | (0.014) | (0.015) | (0.016) |  | (0.047) | (0.051) | (0.049) | |  | | (0.111) | | (0.119) | | (0.126) | | |
| Birth Order (Ref: 5th or higher) | |  |  |  |  |  |  | |  | |  | |  | |  | | |
| First | -0.025 | -0.024 | -0.024 |  | 0.050 | 0.054 | 0.05 | |  | | 0.255** | | 0.291* | | 0.278* | | |
|  | (0.037) | (0.036) | (0.036) |  | (0.053) | (0.055) | (0.055) | |  | | (0.094) | | (0.109) | | (0.104) | | |
| Second-Fourth | -0.034 | -0.033 | -0.033 |  | 0.03 | 0.029 | 0.027 | |  | | 0.258*** | | 0.284*** | | 0.274*** | | |
|  | (0.026) | (0.025) | (0.025) |  | (0.045) | (0.050) | (0.050) | |  | | (0.053) | | (0.065) | | (0.063) | | |
| Child's Sex: Boy | 0.011 | 0.011 | 0.010 |  | -0.005 | 0.007 | 0.004 | |  | | 0.025 | | 0.060 | | 0.061 | | |
|  | (0.011) | (0.011) | (0.011) |  | (0.017) | (0.021) | (0.021) | |  | | (0.040) | | (0.044) | | (0.046) | | |
| Born in Facility | -0.007 | -0.001 | 0.000 |  | 0.119*** | 0.100*** | 0.098*** | |  | | 0.105* | | 0.028 | | 0.022 | | |
|  | (0.016) | (0.016) | (0.016) |  | (0.024) | (0.025) | (0.025) | |  | | (0.048) | | (0.041) | | (0.039) | | |
| Mother's Age | -0.002 | -0.002 | -0.002 |  | -0.001 | 0.000 | 0.000 | |  | | 0.016*** | | 0.015** | | 0.014** | | |
|  | (0.001) | (0.001) | (0.001) |  | (0.003) | (0.003) | (0.003) | |  | | (0.003) | | (0.005) | | (0.005) | | |
| Mother's Education (Ref: No School) | | |  |  |  |  |  | |  | |  | |  | |  | | |
| Primary School | -0.032 | -0.031 | -0.031 |  | 0.004 | -0.007 | -0.007 | |  | | -0.023 | | -0.060 | | -0.059 | | |
|  | (0.018) | (0.017) | (0.018) |  | (0.025) | (0.026) | (0.027) | |  | | (0.043) | | (0.054) | | (0.052) | | |
| Secondary School | -0.056* | -0.051 | -0.051 |  | 0.183*** | 0.204*** | 0.207*** | |  | | -0.001 | | 0.002 | | 0.005 | | |
|  | (0.025) | (0.024) | (0.024) |  | (0.048) | (0.048) | (0.049) | |  | | (0.111) | | (0.131) | | (0.129) | | |
| Mother Married | -0.03 | -0.033 | -0.032 |  | -0.039 | -0.032 | -0.034 | |  | | 0.019 | | 0.035 | | 0.027 | | |
|  | (0.020) | (0.019) | (0.019) |  | (0.044) | (0.056) | (0.056) | |  | | (0.067) | | (0.062) | | (0.061) | | |
| Improved Sanitation | 0.008 | 0.006 | 0.014 |  |  |  |  | |  | |  | |  | |  | | |
|  | (0.019) | (0.020) | (0.018) |  |  |  |  | |  | |  | |  | |  | | |
| Clean Water Source | -0.024 | -0.026 | -0.027 |  |  |  |  | |  | |  | |  | |  | | |
|  | (0.027) | (0.029) | (0.028) |  |  |  |  | |  | |  | |  | |  | | |
| Slept under bednet | 0.007 | 0.008 | 0.008 |  |  |  |  | |  | |  | |  | |  | | |
|  | (0.014) | (0.015) | (0.015) |  |  |  |  | |  | |  | |  | |  | | |
| Previous Child Death |  |  |  |  | 0.052 | 0.048 | 0.048 | |  | | 0.137** | | 0.154* | | 0.155* | | |
|  |  |  |  |  | (0.033) | (0.037) | (0.037) | |  | | (0.045) | | (0.060) | | (0.057) | | |
| Constant | 0.446*** | 0.470*** | 0.455*** |  | 0.099 | 0.187 | 0.192 | |  | | -0.071 | | 0.078 | | 0.139 | | |
|  | -0.075 | -0.071 | -0.074 |  | (0.125) | (0.131) | (0.132) | |  | | (0.186) | | (0.196) | | (0.182) | | |
| Number of Clusters | 150 | 150 | 150 |  | 150 | 150 | 150 |  | | 150 | | 150 | | | | 150 |  |
| Number of Children | 4501 | 4501 | 4501 |  | 1355 | 1355 | 1355 |  | | 399 | | 399 | | | | 399 |  |

* p<0.05, **p<0.01, *** p<0.001
